# Complete opioid transition to sublingual Buprenorphine after abdominal surgery is associated with significant reductions in opioid requirements, but not reduction in hospital length of stay: a retrospective cohort study

**DOI:** 10.1186/s12871-021-01531-2

**Published:** 2022-01-21

**Authors:** Charlotte Heldreich, Sameer Ganatra, Zheng Lim, Ilonka Meyer, Raymond Hu, Laurence Weinberg, Chong O. Tan

**Affiliations:** 1 Anaesthesia, Pain and Perioperative Medicine, Austin Hospital, Level 2 Austin Towers, 145 Studley Rd Heidelberg, Melbourne, VIC 3084 Australia; 2 The Whittington Hospital, Magdala Avenue, Highgate, London, N19 5NF UK

## Abstract

**Background:**

The use of sublingual buprenorphine (SLBup) for acute pain after major abdominal surgery may offer the potential advantages of unique analgesic properties and more reliable absorption during resolving ileus. We hypothesized that complete opioid transition to SLBup rather than oral oxycodone (OOxy) in the early postoperative period after major abdominal surgery would reduce hospital length of stay, and acute pain and total OMEDD (Oral Morphine Equivalent Daily Dose) requirements in the first 24 h from post-parenteral opioid transition.

**Methods:**

We reviewed 146 patients who had undergone elective and emergency abdominal surgery under our quaternary referral centre’s Upper Gastro-Intestinal and Colo-Rectal Surgical Units 6 months before and after the introduction of complete postoperative transition to sublingual buprenorphine, rather than oral oxycodone, in July 2017. Our primary endpoint was 24-hourly post-transition OMEDDs; secondary endpoints were 24-hourly post-transition Mean NRS-11 pain scores on movement (POM) and length of hospital stay (LOS). Univariate analysis and linear multivariate regression analyses were used to quantify effect size and identify surgical, patient & other analgesic factors associated with these outcome measures.

**Results:**

Patients transitioning to SLBup had reduced 24-hourly post-transition OMEDD requirements on postoperative day 2 (POD) (26 mg less, *p* = 0.04) and NRS-11 POM at POD1 (0.7 NRS-11 units less, *p* = 0.01). When adjusting for patient, surgical and special analgesic factors, SLBup was associated with a similar reduction in OMEDDs (Unstandardised beta-coefficient -26 mg, *p* = 0.0001), but not NRS-11 POM (*p* = 0.47) or hospital LOS (*p* = 0.16).

**Conclusions:**

Our change of practice from use of OOxy to SLBup as primary transition opioid from patient-controlled analgesia delivered full opioid agonists was associated with a clinically significant decrease in 24-hourly post-parenteral opioid transition OMEDDs and improved NRS-11 POM, but without an association with hospital LOS after major abdominal surgery. Further prospective randomized work is required to confirm these observed associations and impact on other important patient-centred outcomes.

## Introduction

Major abdominal surgery carries the risk of persistent postoperative pain in 5–50% of cases [1]. Moreover, in cases of prolonged ileus, the potential for a protracted exposure to significant doses of lipid-soluble opioids may be associated with the development of opioid tolerance [2]. Concurrently, many postoperative patients may find themselves discharged on large doses of opioids for ongoing analgesia, with a proportion continuing such drug therapy because of poorly managed pain, opioid tolerance or dependence, or simply poor opioid stewardship [3]. The resulting complications are part of the 240% increase in hospitalisations and deaths in Australia related to opioid over-use since 2000 [4].

The use of buprenorphine in the treatment of acute pain has enjoyed a growing resurgence in perioperative pain management [5]. Current evidence suggests sublingual or intravenous (IV) buprenorphine has a similar analgesic and side effect profile to parenteral morphine at equipotent doses when treating acute post-surgical pain [6]. In addition, the sublingual route of administration is appealing in this context because of its ease of administration, lack of reliance on patient-controlled analgesia techniques and associated equipment, and rapid and reliable absorption. Furthermore, buprenorphine may also offer unique advantages over other commonly used opioids for postoperative pain management. It is anti-hyperalgesic [7], has a long duration of action, and as a less lipid-soluble agent with slower onset of action, may have a lower potential for developing dependence and tolerance. Finally, opioid antagonists have been reported to shorten ileus and hospital length of stay (LOS) after abdominal surgery [8].

Given that the application of IV opioid patient-controlled analgesia (PCA) techniques, with concurrent use of regional analgesia, neuraxial opioids or ketamine infusions are commonplace after major surgery, the use of buprenorphine is an attractive analgesic alternative. However, when transitioning postoperatively from PCA to oral analgesia in the context of major upper gastrointestinal or colorectal surgery, it is unknown whether sublingual buprenorphine (SLBup) offers improved analgesia and reduced oral morphine equivalent drug dose (OMEDD) requirements compared to orally administered oxycodone (OOxy). We primarily hypothesised that use of SLBup would be associated with significant reductions in post-parenteral OMEDD requirements compared with OOxy. Our secondary hypotheses were that use of SLBup would be associated with improved analgesia and reduced hospital LOS without any increase in opioid related critical incidents. We conducted a retrospective study to evaluate these hypotheses and inform our institution whether a prospective randomised trial is justified.

## Methods

Following Human Research Ethics Committee approval (LNR/19/Austin/35), we performed a single centre retrospective cohort study of patients who underwent elective and emergency upper gastrointestinal and colorectal surgery between July 2016 – July 2019. Austin Health is a quaternary surgical service which undertakes > 150 major abdominal resections annually. Inclusion criteria included adult patients (age > 18 years) undergoing surgery of greater than two hours duration, requiring at least one overnight hospital received stay, and who received opioid patient-controlled analgesia (PCA) as part of their postoperative analgesic strategy. Both open and laparoscopic approaches were included. We excluded patients who had emergency surgery due to trauma, those with opioid tolerance (patients taking, for a week or longer, at least 60 mg of morphine daily, oral oxycodone 30 mg/day, transdermal fentanyl 25 mug/hr., 8 mg of hydromorphone, or an equianalgesic dose of another opioid), and patients who received percutaneous jejunal feeding. Given the retrospective nature of the study, the ethics committee waived the need for patient consent.

All surgeries were performed by specialist gastrointestinal surgeons and all patients underwent a standardised enhanced recovery after surgery program that included preoperative optimisation of comorbidities if required and prehabilitation, standardised intraoperative anaesthesia with goal directed therapy to optimise fluid intervention and guide the use of vasoactive medications, and a standardised postoperative care pathway, which included daily physiotherapy and acute pain service reviews. In the absence of contraindications, all patients received paracetamol and non-steroidal anti-inflammatory medications as part of a multimodal analgesic approach.

Two independent investigators extracted data from Austin Health Cerner® electronic medical records, which allows comprehensive electronic data capture and retrieval of patient health information in the perioperative setting. This study is reported in accordance with Strengthening the Reporting Of Cohort Studies in Surgery (STROCSS) guidelines [9]. Collected data included patient demographics, 11-point pain Numerical Rating Scores (NRS-11 scores), oral morphine equivalent daily dose (OMEDD) data and hospital length of stay.

In July 2017, the Austin Health Acute Pain Service initiated a practice change that transitioned patients from a postoperative regime of OOxy to a protocol using SLBup exclusively. Between July 2016 and July 2017 patients were transitioned to pro re nata (prn) OOxy immediate-release from opioid PCA therapy at 50% of the equianalgesic dose and frequency relative to their opioid PCA use over the preceding 24 h. This was done once patients required less than 150 OMEDDs by PCA in the last 24 h and were transitioned from nil-by-mouth to oral sips of fluid following surgical and dietetics review. Transition timing was a clinical decision based on multiple factors, including observed reduction in nasogastric (NG) fluid drainage, reduced abdominal distension, the appearance of bowel sounds or flatus, and absence of associated nausea and vomiting.

Patients receiving SLBup were transitioned from opioid PCA to SLBup at 50% of the equianalgesic dose and frequency relative to their opioid PCA use in the preceding 24 h, with transition timing determined by the same PCA OMEDD threshold as those receiving OOxy, and clinical indicators for the resumption of oral medication.

### Outcomes

Pain endpoints were assessed by taking the mean score derived from the 11-point Numerical Rating Score  (NRS-11) per 24-h period on movement. Pain on movement was defined as the NRS-11 rating on attempted deep breathing and coughing. The outcome measure for our primary hypotheses was total opioid analgesics delivered in the 24 h post-transition, compared to the 24 h period pre-transition, for those transitioned to SLBup compared to OOxy, and converted to OMEDDs as per the Australian & New Zealand College of Anaesthetists & Faculty of Pain Medicine Official Conversion Table 2019 [10]. All opioids administered via all routes (intravenous, transdermal, oral, sublingual) from the time of the arrival to the recovery room, to the last dose of any opioid analgesic administered during the patients hospital stay, were summated and converted to OMEDDs as per the ANZCA conversion rates and included in the analysis if administered within the 24-h pre- and post-transition time. Our secondary outcome measures were:

1. Pain improvement 24 h post-transition, compared to the 24 h period pre-transition, for those transitioned to SLBup compared to OOxy. This was quantified by both the absolute value of NRS-11 scale units in the 24 h post-transition period as well as the fall in NRS-11 scale units in this period as defined by

$$\left[\left( mean\ NRS-11\left[24\right] pre- transition\right)-\left( mean\ NRS-11\left[24\right]\ post- transition\right)\right]$$

(where NRS-11[24] = NRS-11 score over a 24-h period)

Other secondary outcome measures assessed were frequency of pain or opioid-related Medical Emergency Team (MET) calls and hospital length of stay. Severe adverse pain or opioid-related outcomes were defined a priori as any of the following events that occurred in the 48-h period following OOxy or SLBup administration: i) thresholds of hypoxia or tachycardia where pain was deemed to be a causative factor, ii) reduced conscious state (sedation scores of 3 as per the Modified McIntyre Sedation Scale (Appendix 1c) [11]), and iii) a low respiratory rate (≤ 12 breaths/minute).

To account for the effects of the acuity of surgical presentation and the size and location of the surgical incision on our primary and secondary outcome measures, we used arbitrary ordinal scales to stratify the magnitude of these factors (Appendix 1a and b, Table 2). We included single dose pre-operative intrathecal morphine, postoperative ketamine infusion, and regional analgesia (transversus abdominis plane analgesia; rectus sheath or wound catheter analgesia) under the category of special analgesia techniques (SAT) to account for the effects of these techniques on our outcome measures. The postoperative day at which opioid transition occurred was included in the multivariate regression model to account for the natural history of improved pain and reduced OMEDDs as patients recovered from surgery. Regardless of whether the transition was conducted before ‘oral sips’ of fluid was permitted by the surgical team, the duration of postoperative ileus was defined as the number of postoperative days until full ward diet was commenced.

### Statistical methodology

All calculations were performed using SPSS V21 (IBM, New York, USA) statistical software and graphs were prepared with Graph Pad Prism 5 (Graph Pad Software, California, USA). OMEDD data and hospital LOS continuous data were assessed for normality by histogram frequency distribution analysis and the Kolgomorov-Smirnoff normality test, with corresponding parametric descriptive and inferential tests used (mean, SD, unpaired 2-tailed t-tests). Bonferroni correction was applied to univariate analyses where multiple comparisons were used for the same endpoint (POD 1–4 NRS-11 and OMEDDs, *P*-values < 0.0125 considered statistically significant); otherwise *p* <  0.05 was considered statistically significant. To examine the effect of confounding factors on our primary and secondary outcome measures, we used multivariate linear regression to account for the relative impact of patient factors (age, 24-h pre- and post-transition OMEDDs and NRS-11 pain rating assessments), SAT (regional analgesia, intrathecal opioid, ketamine infusions), emergency or elective surgery, ordinal scale of the expected degree of postoperative pain by surgery/incision type and ordinal scale of acuity of surgical presentation type (see appendices A and B) as well as post-transition opioid type. Analyses for collinearity were applied to ensure no dependence between covariates. A priori sample size was determined conservatively by the approximate requirement for 104 samples plus *n* samples for each covariate used in our multivariate linear regression model [12], with the planned inclusion of a maximum of 13 covariates. Covariates were selected for inclusion in each model based on clinical relevance to the dependent variable of interest.

## Results

Over the observation period, 167 patients underwent emergency and elective abdominal surgery under the care of the upper gastrointestinal and colorectal surgery units. One patient was excluded due to opioid tolerance, and 20 patients excluded who received epidural analgesia. A total of 146 patients were included in the final analysis, 82 (56%) receiving OOxy and 64 (44%) receiving SLBup. Patients transitioned to SLBup were more likely to be of higher ASA grade, more painful operation type, have received emergency surgery and more acute surgical presentation, and received open laparotomy. They were also more likely to have reached full ward diet later, and had longer length of stay. Patient, surgical and special analgesic categorical data are presented in Table 1.

**Table 1:** Patient, surgical & analgesic demographics

		OOxy (***N*** = 82)	SLBup (***N*** = 64)	***p***-value
**Age (years)**		60 (17)	65 (13)	0.05
**ASA grade**	**2**	41 (50%)	21 (33%)	0.009
	**3**	37 (45%)	36 (56%)	
	**4**	4 (5%)	7 (11%)	
**Sex (male)**		36 (44%)	26 (40%)	0.61
**Incisional Pain ordinal scale** ^***a***^ **(1–3)**	**1** **2** **3**	22 (27%) 11 (13%) 49 (60%)	36 (56%) 16 (25%) 12 (19%)	0.001
**Emergency surgery**		28 (34%)	32 (50%)	0.003
**Acuity of Surgical Presentation ordinal scale**^***b***^ **(1–5)**	**1** **2** **3** **4** **5**	9 (3%) 10 (8%) 41 (49%) 11 (20%) 11 (20%)	2 (1%) 9 (8%) 24 (33%) 14 (26%) 15 (32%)	< 0.0001
**Postoperative day transitioned to Oxy or SLBup**	**1** **2** **3** **4** **> 4**	29 (35%) 28 (34%) 13 (16%) 8 (10%) 4 (5%)	16 (25%) 20 (31%) 14 (22%) 2 (3%) 12 (19%)	< 0.0001
**POD at FWD**	**1** **2** **3** **4** **5** **6** **7** **> 7**	6 (8%) 26 (34%) 22 (29%) 14 (18%) 4 (5%) 3 (4%) 1 (1%) 0 (0%)	2 (4%) 4 (8%) 7 (13%) 5 (10%) 12 (23%) 4 (8%) 5 (10%) 13 (24%)	< 0.0001
**Duration (Days, POD sips to POD of FWD)**		2 (1.2)	3.6 (2.9)	< 0.0001
**Length of hospital stay (days)**		10 (15)	19 (16)	0.001
**Special analgesia techniques (see text)**		34 (41%)	25 (39%)	0.8
**Laparoscopic vs Open Surgery (Lap %)**		49 (60%)	12 (19%)	< 0.0001

Data presented as mean (standard deviation), mean (SD) or number (proportion)

*ASA* American Society of Anaesthesiologists; *FWD* full ward diet; *OOXY* oral oxycodone; *POD* postoperative day; *SLBup* sublingual buprenorphine

### Outcomes

**Fig. 1:**
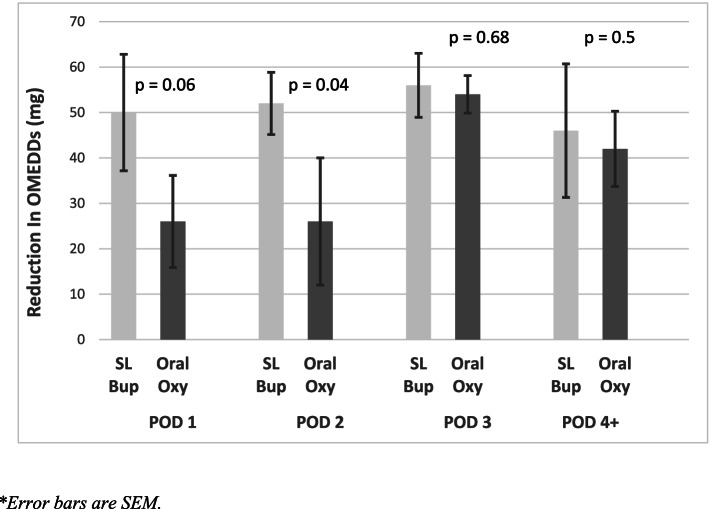
24-hourly post-transitional omedd reduction

**Fig. 2:**
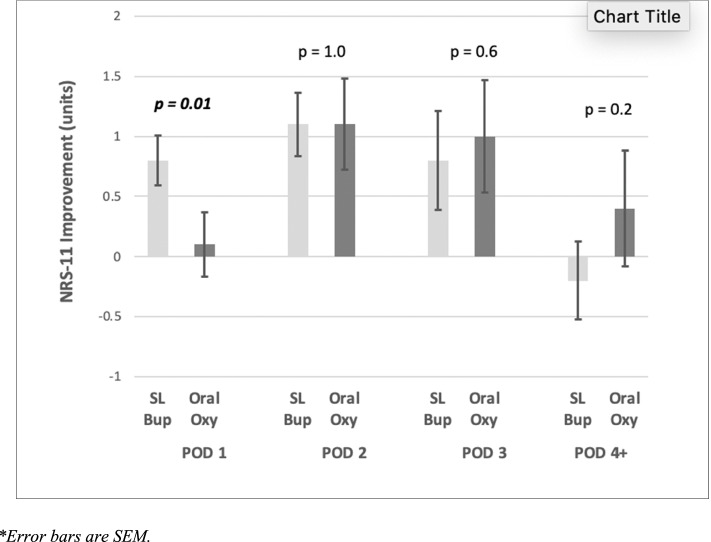
24-hourly post-transitional nrs-11 score improvement on movement

On univariate analysis, patients transitioning to SLBup achieved clinically significant reductions in 24-hourly post-transition OMEDD requirements on postoperative day (POD) 1 and 2 (mean difference 26 & 24 mg, *p* = 0.04 & *p* = 0.06 respectively) that did not reach Bonferroni statistical significance, when compared against those who had transitioned to OOxy. SLBup patients did achieve statistically significant improvements in 24-hourly post-transition NRS-11 pain assessments on movement (POM) on POD 1 (mean difference 0.7 NRS-11 units, *p* = 0.01). The 24-hourly post-transition OMEDD reductions and NRS-11 pain assessment on movement improvements are shown in Figs. 1 and 2.

**Fig. 3:**
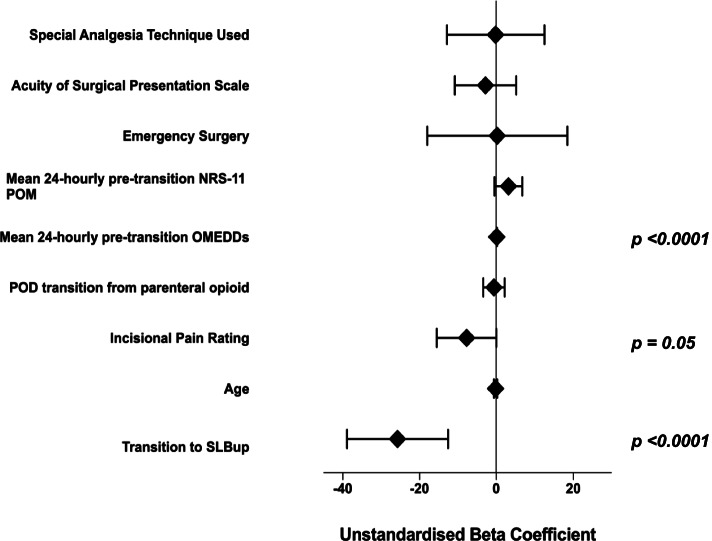
Multivariate linear regression model (dependent variable: 24-hourly post-transition omedds [mg])

 On multivariate linear regression analysis with post-parenteral opioid transition 24 hourly OMEDDs as the dependent variable, patients who had received SLBup showed a statistically and clinically significant reduction in 24 hourly OMEDDs against those who had received OOxy (unstandardised beta-coefficient − 26 mg/day for those receiving SLBup, p = < 0.0001). This result was found after adjusting for the POD at which transition was conducted, surgical factors (incisional pain scale, emergency surgery and surgical acuity presentation scale), and other special analgesic techniques (SAT). POD of analgesic transition did not affect the total post-transition 24-hourly OMEDDs. The multivariate linear regression analysis of the covariates included in the regression model is shown in Figure 3 and Appendix 2(a), Table 3.

**Fig. 4:**
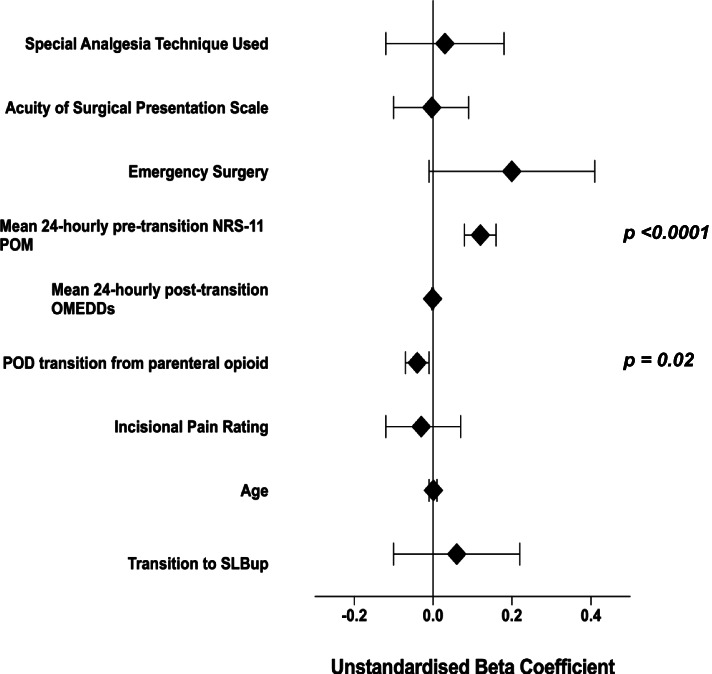
Multivariate linear regression model (dependent variable: 24-hourly post-transition nrs-11 pain on movement, improvement [units])

On multivariate linear regression analysis with post-parenteral opioid transition 24-hourly NRS-11 pain improvement on movement as the dependent variable, there was no association with lower 24-hourly post-transition NRS-11 pain on movement (POM) in patients who had received SLBup over OOxy (*p* = 0.32). This result was found after adjusting for the same surgical, pain, analgesic factors and clinical markers previously outlined. Later POD of analgesic transition worsened post-transition NRS-11 POM (− 0.04 NRS–11 POM units per POD transition day, *p* = 0.02). The multivariate linear regression analysis of the covariates included in this regression model is shown in Fig. 4 and Appendix 2(b), Table 4.

**Fig. 5:**
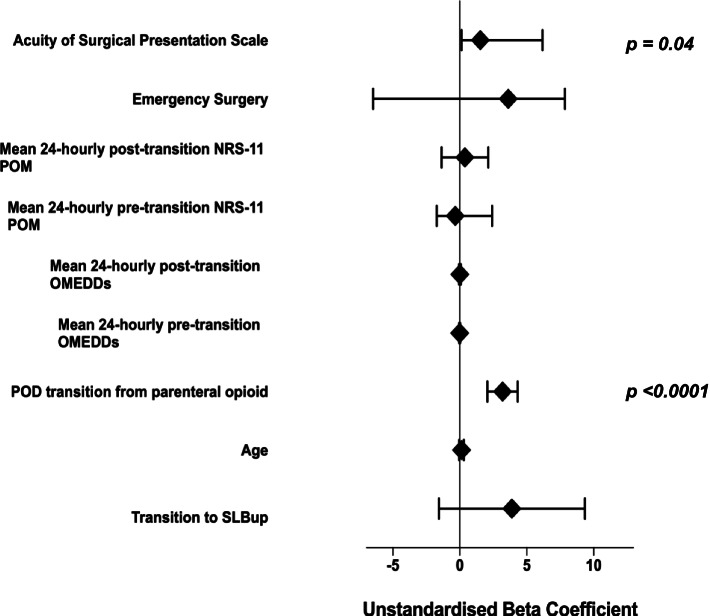
Multivariate linear regression model (dependent variable: hospital length of stay [days])

On multivariate linear regression analysis with hospital LOS as the dependent variable, and after adjusting for the same surgical, pain, analgesic factors and clinical markers as above, complete postoperative transition to SLBup over OOxy had no significant effect on hospital LOS (*p* = 0.16). The multivariate linear regression analysis of the covariates included in this regression model is shown in Fig. 5 and Appendix 2(c), Table 5.

Of note was a 92-year-old patient who had previously undergone an emergency infra-umbilical laparotomy and small bowel resection for bowel obstruction. The patient required administration of IV naloxone after a MET call for a respiratory rate of nine on day two post-surgery, the same day as complete transition to SLBup. A single 200 μg SLBup prn dose had been administered at 0900 h and second at 1900 h, with MET threshold criteria reached for respiratory rate at 2030 h. 50 μg IV fentanyl had been self-administered by a PCA device since 0000 h on the day of transition and ceased at 0800 h, with the patient’s prior 24-hourly fentanyl PCA requirement being 300 μg. No other opioids had been administered, and the respiratory rate rapidly normalised to 18 breaths per minute after a single 40 μg dose of naloxone. There were no other MET calls for pain or opioid-related presentations for any other study patients within 3 days of a complete transition to SLBup or OOxy.

## Discussion

We performed a retrospective cohort study of patients undergoing major abdominal surgery and found that patients receiving SLBup had significantly less 24-hourly OMEDD requirements with improvement in 24-hourly analgesia on movement, without any opioid-related adverse effects. Moreover, our findings are reinforced by the observation the greatest difference in OMEDD reductions were observed at POD 1 and 2 . These results on univariate analyses were found despite patients receiving complete transition to SLBup being more likely to have surgical factors predisposing them to greater postoperative pain and OMEDD requirements.

Buprenorphine’s favourable pharmacodynamic properties have long been recognised as useful in the management of chronic pain and opioid replacement therapy. The medication has low addiction potential [13], has anti-hyperalgesic [7] and anti-neuropathic [14] properties and is purported to have a lower incident rate of respiratory depression compared with other opioid classes [15]. Initial resistance to the adoption of buprenorphine as part of routine acute pain management practice has previously been based on the doubt that, as a partial agonist, its analgesic effect was limited when compared to other full opioid agonists. Contemporary randomised data have dispelled this concern, with equivalent analgesic efficacy having been demonstrated across a range of differing acute pain contexts [16, 17].

More recent studies have not supported the hypothesis that there is a lower risk of respiratory depression associated with buprenorphine [6]. Indeed, the only pain or opioid-related MET call in our study sample was in a patient receiving a very low dose of SLBup, albeit in a patient whose advanced age was a risk factor for opioid sensitivity. Opioid antagonists have demonstrated efficacy in reducing postoperative ileus time [18]. As a partial opioid agonist, buprenorphine may offer similar benefits; however, any effect of buprenorphine over oxycodone on shortening ileus time did not affect hospital LOS in our study. Although none of our results exhibits collinearity that would suggest interdependence within the covariates we analysed, our inpatient pain service’s protocol of attempting opioid transition only when 24-hourly PCA OMEDD use is < 150 mg implies that pain-related surgical or patient factors may account for a later opioid transition.

Our findings imply that SLBup is an efficacious analgesic in the context of postoperative pain management after major abdominal surgery. On univariate analysis, although OMEDD reduction when transitioning from IV opioid PCA to SLBup compared to OOxy did not reach Bonferroni statistical significance on POD 1 and 2, a clinically significant trend was displayed (Fig. 1). Indeed, SLBup patients achieved Bonferroni-corrected statistically significant improvements in 24-hourly post-transition NRS-11 pain assessments on movement on POD 1 (Fig. 2). Moreover, on multivariate analysis patients receiving SLBup showed a statistically and clinically significant reductions in 24 hourly OMEDDs compared to OOxy, reaffirming that compared to OOxy, SLBup may confer significant analgesic clinical benefits, without associated harm. Multivariate analysis did not support an association of SLBup with reduction in NRS-11 POM however, suggesting that surgical and patient factors mitigate any additional analgesic benefit of SLBup in the context of lower OMEDD requirements.

Our study is limited by several factors. As a retrospective cohort study, our findings should be considered associative and hypothesis-forming, with prospective randomised data required to draw causative conclusions. The retrospective nature of our data collection introduces many potential sources of bias and error. Our use of arbitrary incisional pain rating and acuity of surgical presentation ordinal scales is unvalidated. However, to our knowledge, no equivalent validated scales exist in the literature, and these scales were required to account for the effect that clinically important covariates may have on our outcomes of interest. While we have demonstrated an association between clinically important acute pain and opioid requirements, other clinically important secondary patient-centric outcomes, including postoperative complications, inpatient/post-discharge mortality, persistent postoperative pain and long-term opioid use, were not assessed. While our use of multivariate linear regression falls within accepted recommendations for the number of covariates to avoid overfitting in our modelling [19], the relatively small sample size may still lead to Type 1 or Type 2 errors in the results. We limited the maximum number of covariates included in our regression model to 9 to minimise overfitting; however, the nature of the most clinically relevant covariates for each outcome measure resulted in slightly different covariates analysed between models. We specifically did not analyse for opioid side effects of nausea and vomiting in our cohort, as these endpoints would be significantly confounded by postoperative surgical ileus and the intra-abdominal nature of the surgery in our patient sample.

## Conclusions

In a retrospective cohort study of 146 patients undergoing major abdominal surgery, when transitioning from full opioid agonists delivered by PCA devices to oral oxycodone or sublingual buprenorphine, patients receiving sublingual buprenorphine experienced clinically but not statistically significant reductions in post-parenteral OMEDD requirements compared to those receiving oral oxycodone. The same patients experienced a clinically and statistically significant improvement in post-transition analgesia. After adjusting for surgical, patient and analgesic factors, use of sublingual buprenorphine was associated with reduced post-transition OMEDDs, and was not associated with inferior analgesia or a reduction in hospital length of stay. Further prospective randomised studies are justified to confirm these associations.

## Data Availability

The datasets used and/or analysed during the current study are available from the corresponding author on reasonable request. Restrictions apply to the availability of these data, as it is the Austin Hospital policy not to make outcome data for hospital patients freely available, and so will not be made publicly available. Data are however available from the authors upon reasonable request and with permission of Austin Hospital administration.

## Appendix 1

### Appendix 1a – *Ordinal Incisional Pain Rating Scale*

1 = laparoscopic or laparoscopic assisted incision.

2 = Mini- Laparotomy or open incision < 7 cm.

3 = Laparotomy or upper abdominal incision > 7 cm.

### Appendix 1b

**Table 2:** Ordinal Surgical Acuity of Presentation Scale

1 = Elective hernia or equivalent elective abdominal surgery	
2 = Gastric non-cancer surgery not qualifying for higher grade; cholecystitis	
3 = Undifferentiated abdominal pain; elective cancer surgery	
4 = Acute gastro-intestinal obstruction	
5 = Bowel ischaemia or perforated viscus	

### Appendix 1c – *Modified McIntyre Sedation Scale*

0 = Awake and alert.

*S = Patient asleep but rousable.

1 = Easily rousable.

2 = Persistently drowsy, unable to stay awake spontaneously > 10 s.

3 = Difficult to rouse or unresponsive.

## Appendix 2

**Table 3:** Multivariate Linear Regression Model (Dependent Variable: 24-hourly post-transition OMEDDS)

	Unstandardised Beta Coefficients^	***P*** value	95% Confidence Interval for Beta	
			Lower Bound	Upper Bound
**(Constant)**	56.58	0.017	10.19	102.97
**Transition to SLBup** ^b^	−25.73	**< 0.0001**	− 38.90	−12.56
**Age**	−0.15	0.47	−.57	.26
**Incisional pain rating**	−7.70	0.05	−15.47	.07
**POD transition from parenteral opioid**	−0.58	0.69	−3.36	2.20
**Mean 24-hourly pre-transition OMEDDs**	0.16	< 0.0001	.09	.23
**Mean 24-hourly pre-transition NRS-11 POM**	3.19	0.08	−.415	6.803
**Emergency surgery** ^b^	.25	.978	−17.96	18.48
**Acuity of surgical presentation scale**	−2.79	.49	−10.80	5.22
**Special analgesia technique used** ^b^	−.17	.98	−12.86	12.53

#unstandardized coefficients – Covariates (a, b, c etc) affect the dependent variable (y) by Y = constant + (a) x [coefficient] + (b) x [coefficient]

^b^signifies binary covariate – If binary factor is present, there is an increase of (β-coefficient x dependent variable unit) in the dependant variable unit value. For negative β-coefficients, the dependant variable similarly decreases

**Table 4:** Multivariate Linear Regression Model (Dependent Variable: 24-hourly post-transition NRS-11 pain on movement, improvement [%])

	Unstandardised Beta Coefficients^**a**^	***P*** value	95% Confidence Interval for Beta	
			Lower Bound	Upper Bound
**(Constant)**	0.28	0.32	−0.27	−0.82
**Transition to SLBup** ^b^	0.06	0.47	−0.10	0.22
**Age**	0.001	0.89	−0.01	0.01
**Incisional pain rating**	−0.03	0.59	−0.12	0.07
**P-OD transition from parenteral opioid**	−0.04	.024	−.07	−.01
**Mean 24-hourly pre-transition OMEDDs (?delete)**	0.001	.52	−.001	.001
**Mean 24-hourly post-transition OMEDDs**	−0.001	.38	−.003	.001
**Mean 24-hourly pre-transition NRS-11 POM**	0.12	<.0001	.08	.16
**Emergency surgery** ^b^	0.20	0.06	−0.01	0.41
**Acuity of surgical presentation scale**	−0.003	0.95	−0.10	0.09
**Special analgesia technique used** ^b^	0.03	0.67	−0.12	0.18

^**a**^unstandardized coefficients – Covariates (a, b, c etc) affect the dependent variable (y) by Y = constant + (a) x [coefficient_a_] + (b) x [coefficient_b_]

^b^signifies binary covariate – If binary factor is present, there is an increase of (β-coefficient x dependent variable unit) in the dependant variable unit value. For negative β-coefficients, the dependant variable similarly decreases

**Table 5:** Multivariate Linear Regression Model (Dependent Variable: Hospital length of stay)

	Unstandardised Beta Coefficients^a^	*P* value	95% Confidence Interval for Beta	
			Lower Bound	Upper Bound
(Constant)	−15.87	.03	−30.30	−1.44
Transition to SLBup^b^	3.89	.16	−1.56	9.34
Age	0.13	.12	−.04	.30
POD transition from parenteral opioid	3.19	<.0001	2.06	4.32
Mean 24-hourly pre-transition OMEDDs	−0.007	.65	−.04	.02
Mean 24-hourly post-transition OMEDDs	0.002	.95	−.06	.07
Mean 24-hourly pre-transition NRS-11 POM	−0.34	.74	−1.72	2.41
Mean 24-hourly post-transition NRS-11 POM	0.38	.67	−1.36	2.12
Emergency surgery^b^	3.62	.85	−6.48	7.85
Acuity of surgical presentation scale	1.53	.041	.13	6.18

^a^unstandardized coefficients – Covariates (a, b, c etc) affect the dependent variable (y) by Y = constant + (a) x [coefficient_a_] + (b) x [coefficient_b_]

^b^signifies binary covariate – If binary factor is present, there is an increase of (β-coefficient x dependent variable unit) in the dependant variable unit value. For negative β-coefficients, the dependant variable similarly decreases

## Abbreviations

SLBup: Sublingual buprenorphine
OOxy: Oral oxycodone
POD: Postoperative day
POM: Pain on movement
NRS-11: 11-point numerical rating scale (for pain)
LOS: Length of stay
PCA: Patient controlled analgesia
OMEDD: Oral Morphine Equivalent Drug Dose
MET: Medical emergency team
SAT: Special analgesia techniques
NG: Nasogastric
